# Consumption of Cuban Policosanol Improves Blood Pressure and Lipid Profile via Enhancement of HDL Functionality in Healthy Women Subjects: Randomized, Double-Blinded, and Placebo-Controlled Study

**DOI:** 10.1155/2018/4809525

**Published:** 2018-04-16

**Authors:** Kyung-Hyun Cho, Suk-Jeong Kim, Dhananjay Yadav, Jae-Yong Kim, Jae-Ryong Kim

**Affiliations:** ^1^Department of Medical Biotechnology, Yeungnam University, Gyeongsan 712-749, Republic of Korea; ^2^Research Institute of Protein Sensor, Yeungnam University, Gyeongsan 712-749, Republic of Korea; ^3^LipoLab, Yeungnam University, Gyeongsan 712-749, Republic of Korea; ^4^Department of Biochemistry and Molecular Biology, Smart-Aging Convergence Research Center, College of Medicine, Yeungnam University, Daegu 705-717, Republic of Korea

## Abstract

Policosanol has been reported to improve blood pressure, lipid profile, and HDL functionality via inhibition of cholesteryl ester transfer protein (CETP) both *in vitro* and *in vivo* in zebrafish and human models. However, there are limited reports and randomized, double-blinded trials on policosanol that could advocate the blood pressure-lowering effect in prehypertensive participants. Therefore, we performed *in vitro*, *in vivo*, and ex vivo experiments to provide more substantial and concrete data on the blood pressure-lowering effect of policosanol. Consumption of policosanol for 8 weeks enhanced plasma antioxidant activity. In the policosanol group, plasma total cholesterol (TC) and triglyceride (TG) levels were reduced up to 20% and 14%, respectively, and HDL-C level was elevated up to 1.3-fold compared to that at week 0. TG/HDL-C and cholesteryl ester transfer protein (CETP) activities were reduced up to 36% and 20%, respectively. Uptake of oxidized LDL in macrophages was reduced as oxidized species levels were reduced, and HDL_2_-associated paraoxonase activities were enhanced by 60% compared to those at week 0. Encapsulation of policosanol into reconstituted HDL (PCO-rHDL) enhanced cholesterol efflux activity and insulin secretion capacity. In conclusion, consumption of policosanol for 8 weeks in healthy female subjects resulted in lowered blood pressure and CETP activity via elevation of HDL/apoA-I contents and enhancement of HDL functionalities, including cholesterol efflux and insulin secretion. These functional enhancements of HDL can contribute to the prevention of aging-related diseases, hypertension, and stroke.

## 1. Introduction

It is well known that elevation of serum HDL-C levels is an effective strategy for suppressing the incidence of aging-related diseases such as cardiovascular disease (CVD), diabetes, and Alzheimer's disease [1]. In addition to HDL-C quantity, it has been firmly established that HDL quality and functionality are more important in the suppression of aging-related diseases [2]. However, there has been no strategy involving the use of dietary foods or medicines in the elevation of HDL-C quantity and enhancement of HDL functionality except for aerobic exercise [3]. As a functional food, Cuban policosanol (PCO) was reported to elevate HDL-C levels in hypercholesterolemic rabbits and humans as well as reduce LDL-C levels and oxidation [4–6].

Policosanol enhances the beneficial functions of HDL and maximizes its antioxidant, antiglycation, and antiatherosclerotic activities along with inhibition of CETP activity [7–9]. These results suggest an association between policosanol activity and HDL functionality for enhancement of longevity. Reconstituted HDL (rHDL) containing policosanol (PCO-rHDL) was shown to induce upregulation of tissue regeneration activity in a zebrafish model [7] along with a lipid-lowering effect [8]. However, until now, the basic mechanism of policosanol has not been fully elucidated. One problematic hurdle preventing any investigation into the physiological functions of policosanol is its water insolubility in enzyme assay, cell-based assay, and *in vivo* animal systems. To overcome this, a policosanol mixture was assimilated into reconstituted HDL with apoA-I in order to investigate the physiological functions of policosanol in lipoprotein metabolism [7]. Policosanol in rHDL has potent antioxidant, antiglycation, and CETP inhibitory activities as well as tissue regeneration activity, especially upon integration into HDL. The physiological effect of policosanol was investigated in brain cells (neuroglioma) and hypercholesterolemic zebrafish. Nine weeks of policosanol consumption resulted in decreased serum TC and TG levels, increased HDL-C levels via CETP activity inhibition, and amelioration of fatty liver [8]. Kaup et al. previously reported that Egyptian rice bran extract, which is enriched with policosanol and *γ*-oryzanol, has an antidiabetic effect in rats [10].

We recently reported that daily consumption of policosanol by young smoker (YS, *n* = 7) and middle-aged male participants (MN, *n* = 11) for 8 weeks resulted in a lowering of systolic blood pressure up to 4%. The serum TG levels exhibited a reduction up to 28 and 26% from the baseline values in the young nonsmoker (YN, *n* = 7) and middle-aged participants. Nonetheless, the percentage of HDL-C in total cholesterol was elevated in all male participants (YN, 36%; YS, 35%; MN, 8%) after 8 weeks of policosanol consumption [9]. Nonetheless, our previous report was a pilot study in a different group of participants and needed a more specific data that may suggest the efficacy of policosanol on blood pressure. Moreover, the study lacks an appropriate control.

Although there have been many conflict data and arguments about the cholesterol-lowering efficacy of policosanol [11, 12], a recent meta-analysis [13] of randomized controlled trials from 22 studies including 1886 subjects concluded that policosanol could significantly reduce total cholesterol and LDL-C and increase HDL-C. In spite of many reports having examined the efficacy of policosanol in human subjects and animal models, there has been no *in vitro* or *in vivo* study on HDL functionality such as enhancement of cholesterol efflux and antioxidant ability with individually purified lipoprotein by sequential density gradient ultracentrifugation rather than concentration measured in the serum sample. Therefore, we tested the physiological effects of policosanol consumption on blood pressure and HDL functionality in healthy Korean female subjects.

## 2. Materials and Methods

### 2.1. Policosanol and Encapsulation

Policosanol tablet (10 mg) was obtained from Rainbow & Nature Pty, Ltd (Thornleigh, NSW, Australia). Policosanol (sugar cane wax alcohol, SCWA) contains several alcohol chains of various lengths. Contents of higher aliphatic alcohols were >90%. The individual alcohols present in policosanol are 1-tetracosanol (C_24_H_49_OH; molecular weight (MW): 354.7 m*μ*) ≤2%, 1-hexacosanol (C_26_H_53_OH; MW: 382.4 m*μ*) ≤4.5–10%, 1-heptacosanol (C_27_H_55_OH; MW: 396.4 m*μ*) ≤5%, 1-octacosanol (C_28_H_57_OH; MW: 410.5 m*μ*) ≤60–70%, 1-nonacosanol (C_29_H_59_OH; MW: 424.8 m*μ*) ≤2%, 1-triacontanol (C_30_H_61_OH; MW: 438.5 m*μ*) ≤10–15%, 1-dotriacontanol (C_32_H_65_OH; MW: 466.5 m*μ*) ≤3–8%, and 1-tetratriacontanol (C_34_H_69_OH; MW: 494.5 m*μ*) ≤2%.

To overcome the insolubility of policosanol in aqueous isotonic buffer, we synthesized rHDL-containing PCO (PCO-rHDL). A rHDL-containing policosanol was synthesized according to our previous report [7] by the sodium cholate dialysis method using initial molar ratios of 95 : 5 : 1 for POPC : cholesterol : apoA-I containing 0.5 *μ*g, 2.5 *μ*g, or 5 *μ*g of policosanol.

### 2.2. Participants

**Figure 1:**
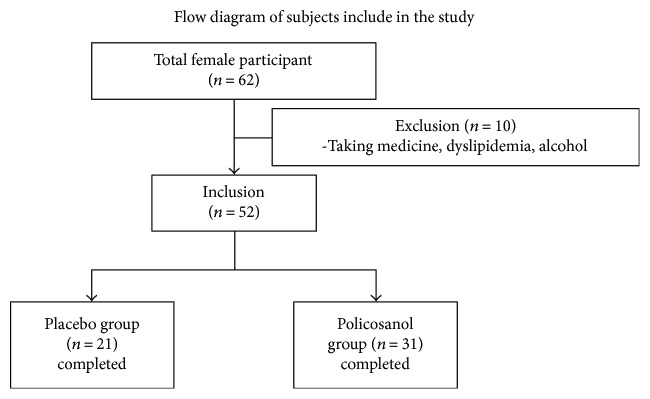
Design of study and participants. Inclusion criteria were normolipidemic, normoglycemic, and healthy subjects. Exclusion criteria were heavy alcohol drinkers (30 g > day of EtOH), patients with endocrinological disorders, and those taking hyperlipidemic medicine.

We recruited healthy female volunteers who had prehypertension (systolic 120–139 mmHg, diastolic 80–89 mmHg). All volunteers were prescreened for eligibility for the following inclusion criteria: age 18–65 years who had prehypertension without any known endocrinological disorder. Heavy alcohol consumers (>30 g EtOH/day) and those who consumed any prescribed drugs for hyperlipidemia, diabetes mellitus, or hypertension were excluded. All subjects had unremarkable medical records without prohibited drug use or history of systemic diseases. On the first visit day, all participants casted dice for randomized grouping. The description of the study is shown in Figure 1, and the recruited participants consumed policosanol for 8 weeks. We analyzed serum parameters from all participants who consumed policosanol daily (10 mg tablet) or placebo for 8 weeks. Informed consent was obtained from all participants prior to commencement of the study, and the Institutional Review Board at Yeungnam University (Gyeongsan, South Korea) approved the protocol (IRB no. 7002016-A-2016-021).

### 2.3. Study Design

This study was a double-blinded, randomized, and placebo-controlled trial with 8-week treatment periods. Subjects were instructed to take one tablet containing policosanol (10 mg of sugar cane wax alcohol) or placebo consisting of a dextrin and lactose mixture, manufactured in Cosmax Bio Inc. (Jecheon, Korea), per day. Other ingredients to make tablet are corn starch, cellulose, gelatin, stearic acid, and so on. All ingredients, the manufacturing process, and the facility were approved by Korean FDA.

All participants were instructed to avoid excess alcohol drinking (less than 30 g of EtOH per day). They were also instructed to avoid vigorous exercise (less than 30 min per day at 60–80% maximum capacity). If subjects had a sedentary lifestyle before enrollment, we recommended them to maintain their lifestyle during the consumption period to avoid bias due to excess exercise.

### 2.4. Anthropometric Analysis

Height, body weight, body mass index (BMI), total body fat (%), total body fat mass (kg), and visceral fat mass (kg) of each participants were measured at the same time of the day at 4-week intervals using an X-Scan Plus II body composition analyzer (Jawon Medical, Gyeongsan, Korea).

### 2.5. Measurement of Blood Pressure

Blood pressure was measured each visit for three times, and the average was recorded at 4-week intervals using three methods. First, we used a digital blood pressure monitor (Omron HBP-9020, Kyoto, Japan). Second, a pulse wave analyzer, SphygmoCor system (AtCor Medical, Sydney, Australia), was used to measure brachial and aortic artery blood pressure. Third, a mercury sphygmomanometer was used for manual measurement by a licensed technician.

To diagnose hypertension, three different methods were employed in the current study to measure blood pressure based on the brachial and radial arteries. However, there exists a discrepancy in blood pressure measurement among these devices [14]. Instruments such as the Omron digital blood pressure device and mercury sphygmomanometer are commonly used in hospitals to estimate blood pressure. However, in epidemiological studies, focusing on central aortic blood pressure is advisable due to its various advantages over traditional blood pressure measurements. Aortic blood pressure is the pressure exerted on the heart and brain and is dissimilar to blood pressure in the limbs such as the arms. Central aortic blood pressure is more accurate and standardized for the diagnosis and management of hypertension compared to blood pressure measurements based on brachial arteries [15]. In comparing their predictive values for cardiovascular mortality, central blood pressure was shown to be better than brachial blood pressure [16]. Brown reported that measurement of blood pressure using a mercury sphygmomanometer or Omron digital machine is based on the brachial artery, which has a structure that is reportedly unaffected by hypertension [17]. To overcome these limitations, this study used three different types of methods for measuring blood pressure.

### 2.6. Measurement of Augmentation Index and Augmentation Pressure

Augmentation index, which is the difference between the second and first systolic peaks expressed as a percentage of the pulse pressure, is a measure of systemic arterial stiffness and wave reflection, as described previously [18]. A licensed technician trained in the technique and blinded to the characteristics of each subject performed measurements of augmentation pressure and augmentation index.

### 2.7. Plasma Analysis

Blood was obtained from the subjects following overnight fasting. Blood was collected using a vacutainer (BD Biosciences, Franklin Lakes, NJ, USA) containing EDTA (final concentration of 1 mM) at weeks 0 and 8 during intake of policosanol. Plasma was isolated by low-speed centrifugation (3000*g*) and stored at −80°C until analysis. To analyze plasma, total cholesterol (TC), triglyceride (TG), high-density lipoprotein cholesterol (HDL-C), glucose, uric acid, aspartate aminotransferase (AST), and alanine aminotransferase (ALT) levels were measured using commercially available kits (Cleantech TS-S; Wako Pure Chemical, Osaka, Japan). Plasma aldosterone levels was measured by radioimmunoassay (RIA) using the instrument 1470-Gamma Counter (PerkinElmer) via Seegene Medical Foundation (Seoul, Korea).

### 2.8. Ferric Reducing Ability of Plasma Assay

The ferric reducing ability of plasma (FRAP) was determined using the method described by Benzie and Strain [19]. The antioxidant activities of individual HDL fractions (20 *μ*g each in PBS) were estimated by measuring increases in absorbance induced by generated ferrous ions.

### 2.9. Characterization of Lipoproteins

Very low-density lipoprotein (VLDL, *d* < 1.019 g/mL), low-density lipoprotein (LDL, 1.019 < *d* < 1.063), high-density lipoprotein_2_ (HDL_2_, 1.063 < *d* < 1.125), and high-density lipoprotein_3_ (HDL_3_, 1.125 < *d* < 1.225) were isolated from the individual plasma of each group via sequential ultracentrifugation [20], and the density was adjusted by addition of NaCl and NaBr in accordance with standard protocols. Samples were centrifuged for 22 hr at 10°C and 100,000*g* using a Himac CP100NX (Hitachi, Tokyo, Japan) at the Instrumental Analysis Center of Yeungnam University. To measure lipoproteins, total cholesterol (TC) and triglyceride (TG) levels were analyzed using commercially available kits (Cleantech TS-S; Wako Pure Chemical, Osaka, Japan). Protein concentrations of lipoproteins were calculated via Lowry protein assay, as modified by Markwell et al. [21].

To estimate the degree of oxidation in lipoprotein, the concentration of oxidized species in lipoproteins was determined by the thiobarbituric acid reactive substance (TBARS) assay method using malondialdehyde as a standard [22]. To differentiate the extent of glycation between the groups, advanced glycation end products (AGEs) in lipoproteins were determined from reading fluorometric intensities at 370 nm (excitation) and 440 nm (emission), as described previously [23], using a spectrofluorometer LS55 (PerkinElmer, Shelton, CT, USA) with the WinLab software package (version 4.0).

### 2.10. Cholesteryl Ester Transfer Protein Assay

A rHDL-containing apoA-I and cholesteryl oleate were synthesized in accordance with the method described by Cho [24] using trace amounts of [^3^H]-cholesteryl oleate (TRK886, 3.5 *μ*Ci/mg of apoA-I; GE Healthcare). Briefly, lipids (POPC, cold cholesteryl oleate, and [^3^H]-cholesteryl oleate) were mixed in a glass vial and gently vortexed, followed by drying under a N_2_ gas stream at 37°C. After drying, the lipids were dispersed by addition of TBS with slight agitation. Phospholipid bilayer formation was facilitated by addition of sodium cholate and apoA-I. After extensive dialysis for 24 hr to remove cholate, [^3^H]-CE-rHDL was recovered and characterized by scintillation counting and protein determination.

[^3^H]-CE-rHDL was immobilized using CNBr-activated Sepharose 4B resin (Amersham Biosciences) for easy separation after the reaction, in accordance with the manufacturer's instructions. CE transfer reaction was performed in 300 *μ*L reaction mixtures containing human serum (20 *μ*L) or HDL_3_ (20 *μ*L, 2 mg/mL) as a cholesteryl ester transfer protein (CETP) source, [^3^H]-rHDL-agarose (20 *μ*L, 0.25 mg/mL) as a CE donor, and human LDL (20 *μ*L, 0.25 mg/mL) as a CE acceptor. After incubation at 37°C, the reaction was halted via brief centrifugation (10,000*g*) for 3 min at 4°C. The supernatant containing the CE acceptor (150 *μ*L) was then subjected to scintillation counting, and percentage transfer of [^3^H]-CE from [^3^H]-rHDL to LDL was calculated.

### 2.11. Paraoxonase Assay

Paraoxonase-1 (PON-1) activity was determined by measuring the initial velocity of *p*-nitrophenol production at 37°C based on its absorbance at 405 nm (microplate reader, Bio-Rad model 680; Bio-Rad, Hercules, CA, USA), as described previously [25] with slight modification [26]. Prior to the measurement, HDL was thoroughly dialyzed against PBS to eliminate EDTA.

### 2.12. LDL Oxidation

Oxidized LDL (oxLDL) was obtained by incubation of the LDL fraction with CuSO_4_ (final concentration of 10 *μ*M) for 4 hr at 37°C. oxLDL was then filtered through a 0.22 *μ*m filter (Millex; Millipore, Bedford, MA) and measured by thiobarbituric acid reactive substances (TBARS) assay to determine the extent of oxidation [22].

### 2.13. Phagocytosis of LDL into Macrophages

THP-1 cells, a human monocytic cell line, were obtained from the American Type Culture Collection (ATCC, TIB-202™, Manassas, VA, USA) and maintained in RPMI 1640 medium (HyClone, Logan, UT) supplemented with 10% fetal bovine serum until needed. Cells below 20 passages were incubated in medium containing phorbol 12-myristate 13-acetate (PMA, 150 nM) in 24-well plates for 48 hr at 37°C in a humidified incubator (5% CO_2_, 95% air) in order to induce differentiation into macrophages. Differentiated and adherent macrophages were then rinsed with warm PBS, followed by incubation with 450 *μ*L of fresh RPMI 1640 medium containing 0.1% FBS and 50 *μ*g of each LDL (1 mg of protein/mL in PBS) for 48 hr at 37°C in a humidified incubator. After incubation, cells were washed with PBS three times and then fixed in 4% paraformaldehyde for 10 min. Next, fixed cells were stained with Oil Red O staining solution (0.67%) and washed with distilled water. THP-1 macrophage-derived foam cells were then observed and photographed using a Nikon Eclipse TE2000 microscope (Tokyo, Japan) at 400x magnification, as in our previous report [27]. Cell medium (0.2 mL) was then analyzed by the TBARS assay to evaluate changes in the levels of oxidized species using a malondialdehyde (MDA) standard.

### 2.14. Antiatherogenic Activity of HDL_3_

Differentiated and adherent macrophages were then washed with warm PBS and incubated with 400 *μ*L of fresh RPMI 1640 medium containing 0.1% fetal bovine serum, 50 *μ*g of oxLDL (1 mg of protein/mL in PBS), and 30 *μ*g of HDL_3_ (2 mg of protein/mL in PBS) from each group for 48 hr at 37°C in a humidified incubator. After incubation, cells were stained with Oil Red O solution (0.67%) to visualize the amount of lipid species in cells. THP-1 macrophage-derived foam cells were then observed and photographed using a Nikon Eclipse TE2000 microscope (Tokyo, Japan) at 400x magnification. Quantification area was carried out via computer-assisted morphometry using Image-Pro Plus software (version 4.5.1.22, Media Cybernetics, Bethesda, MD).

### 2.15. *In Vitro* Cholesterol Efflux

THP-1 cells were incubated in medium containing phorbol 12-myristate 13-acetate (PMA, 150 nM) on a plate for 48 hr at 37°C in a humidified incubator to induce differentiation into macrophages. The macrophages were treated with radiolabeled cholesterol (0.1 *μ*Ci of [^3^H]-cholesterol) in RPMI 1640 medium (HyClone, Logan, UT) containing 1% fetal bovine serum (HyClone, Logan, UT) per well (0.5 mL) for 48 hr. The medium containing the isotope was saved and replaced with fresh media containing 0.3 mM 8-(4-chlorophenylthio)-cyclic adenosine monophosphate (cAMP, Cat. No. C3912, Sigma-Aldrich, St. Louis, MO) for upregulation of cellular cholesterol pump (adenosine triphosphate- (ATP-) binding cassette (ABC) transporter-1, ABCA-1) for 18 hr. After removal of media containing cAMP, human HDL_3_ (28 *μ*g of apoA-I) or rHDL containing policosanol was added and incubated with serum-free media (0.5 mL) for 24 hr. Subsequently, the cell medium (0.5 mL) in individual wells was collected in a 1.7 mL tube. Cells were rinsed with PBS three times and dissolved in 0.2 mL of RIPA buffer (50 mM Tris-HCl [pH 8.0], 150 mM NaCl, 5 mM EDTA [pH 8.0], 1% NP-40, 0.5% sodium deoxycholate, and 0.1% sodium dodecyl sulfate) for cell lysis. An aliquot of the cell lysate (0.1 mL) was mixed with scintillation cocktail (3 mL) to quantify the isotope amount of cholesterol taken up into cells. After scintillation counting of [^3^H]-cholesterol in cells and medium, the amount of effluxed cholesterol from cells was calculated using the following formula [28]:

(1) $$\begin{array}{c} \%Cholesterol\;efflux=\frac{\left(media\;counts\times dilution\;factor\right)}{\left(media\;count\times dilution\;factor\right)+\left(cell\;lysis\;count\times dilution\;factor\right)}\times 100,\\ \%Net\;efflux=\%cholesterol\;efflux\;\left(with\;{HDL}_{3}\right)-\%blank\;efflux\;\left(without\;{HDL}_{3}\right). \end{array}$$

### 2.16. ELISA and Western Blot

To evaluate CETP activity in plasma, each well of a polystyrene microplate (no. 3590; Corning Inc., Corning, NY, USA) was coated with anti-human CETP rabbit antibody (ab19012; Abcam, Cambridge, UK) at a concentration of 0.25 *μ*g/mL and incubated overnight at 4°C. Equally, diluted serum samples were incubated for 2 hr at room temperature. After extensive washing, anti-human CETP mouse antibody (ab2726; Abcam, 1 *μ*g/mL) was treated and incubated for 2 hr at room temperature. To develop the color reaction, anti-mouse IgG antibody (ab6728; Abcam, 0.5 *μ*g/mL conjugated with horseradish peroxidase) was added. For color development, 3,3′,5,5′ tetramethylbenzidine (TMB) substrate solution (Cat. No. 555214; BD Biosciences, Franklin Lakes, NJ, USA) was treated and quantified using a VICTOR X4 microplate reader (Perkin Elmer, Waltham, MA).

Apolipoprotein/lipoprotein constitution was compared via sodium dodecyl sulfate-polyacrylamide gel electrophoresis (SDS-PAGE) with identical protein loading quantities (5 *μ*g of total protein per lane) from cell lysate via immunodetection. Anti-human apoA-I antibody (ab7613), anti-ABCA1 antibody (ab24261), and anti-GAPDH antibody (ab6672) were purchased from Abcam (Cambridge, UK). The relative band intensities were compared via band scanning using a Gel Doc® XR (Bio-Rad, Hercules, CA) with Quantity One software, version 4.5.2. We used simple basic steps to measure the band intensity by densitometry analysis. Blot images are imported into the Quantity One software, and then the contrast was adjusted in such a manner that the bands were clearly noticeable on the blot image. The area around each band was selected; further, the background intensity was subtracted from the blot image. The bands were then outlined by drawing a boundary around it; band intensities were exported in excel format for further analysis.

### 2.17. Insulin Secretion Assay

A rat insulinoma cell line (INS-1), kindly provided by K-C. Won (Department of Internal Medicine, College of Medicine, Yeungnam University), was maintained at 37°C in RPMI 1640 medium (Gibco BRL, Grand Island, NY, USA) containing 11.1 mmol/L of glucose and 2 mmol/L of L-glutamine. The medium was supplemented with 10% FBS, 1 mmol/L of pyruvate, 10 mmol/L of HEPES, 50 *μ*mol/L of *β*-mercaptoethanol, 100 units/mL of penicillin, and 100 *μ*g/mL of streptomycin (INS-1 medium), as in our previous report [29]. INS-1 cells were incubated at 37°C in the presence or absence of PCO-rHDL or plasma HDL. Incubations were carried out under low (final concentration of 2.8 mM) or high glucose concentration (final concentration of 25 mM) in culture medium, as previously reported [30]. After incubation, insulin secretion was determined using a radioimmunoassay kit (rat insulin RI-13K; Millipore, Billerica, MA, USA), according to the manufacturer's recommendation.

### 2.18. Electron Microscopy

Transmitted electron microscopy (TEM) was performed with a Hitachi electron microscope (model H-7600; Ibaraki, Japan) operated at 80 kV, as in our previous reports [31]. VLDL, LDL, and HDL were negatively stained with 1% sodium phosphotungstate (pH 7.4) with a final apolipoprotein concentration of 0.3 mg/mL in TBS.

### 2.19. Data Analysis

All data are expressed as the mean ± SD from the three independent experiments with duplicate samples. Data comparisons were carried out by Student's *t*-test using the SPSS program (version 14.0; SPSS Inc., Chicago, IL, USA). The differences between the means were assessed using Duncan's multiple-range test. Statistical significance was defined as *p* < 0.05.

## 3. Results

### 3.1. Changes in Body Composition

After 8 weeks of policosanol consumption, both groups had the same BMI. However, total body fat mass decreased up to 12% in the policosanol group, whereas the control group showed almost no change in body fat mass, as shown in Table 1. For fat distribution, visceral fat mass was reduced more than subcutaneous fat mass up to 20% in the policosanol group.

### 3.2. Blood Pressure

Based on the three measurements, the policosanol group showed significantly reduced average systolic and diastolic blood pressure levels up to 10% and 14%, respectively, whereas the control group showed similar blood pressure levels during 8 weeks of consumption. The policosanol group showed a significant reduction of augmentation index (AI) and augmentation pressure (AP) up to 57% and 72%, respectively, whereas the control group showed no change after consumption (Table 1).

### 3.3. Plasma Lipid Profile and CETP Activity

As shown in Table 1, the policosanol group showed 19% and 14% reductions in TC and TG levels, respectively, at week 8 compared with week 0, whereas the control group showed no difference. Plasma HDL-C level and percentage of HDL-C in TC were significantly elevated in the policosanol group up to 1.3-fold and 1.6-fold, respectively, compared with those at week 0. Furthermore, the calculated ratio of the TG/HDL-C level in the policosanol group was reduced to 1.4 after 8 weeks, whereas the control group showed no change (around 2.1). The calculated LDL-C level was also reduced in the policosanol group by 35%, whereas the control group showed no change. Before policosanol consumption (at week 0), all groups showed relatively high CETP activity (around 38% CE transfer). After 8 weeks, the policosanol group showed a significant reduction in CETP activity (around 31% CE transfer), whereas the control group showed no change. In addition, the serum CETP amount was reduced in the policosanol group up to 25% compared with that at week 0, whereas the placebo group showed no change. There was no significant change in glucose level in both groups from weeks 0 to 8. Uric acid and aldosterone levels were significantly reduced in the participants who consumed policosanol for 8 weeks (10 mg per day). However, there were no significant changes in uric acid and aldosterone in the placebo group after 8 weeks.

### 3.4. Serum Antioxidant Activity

**Figure 2:**
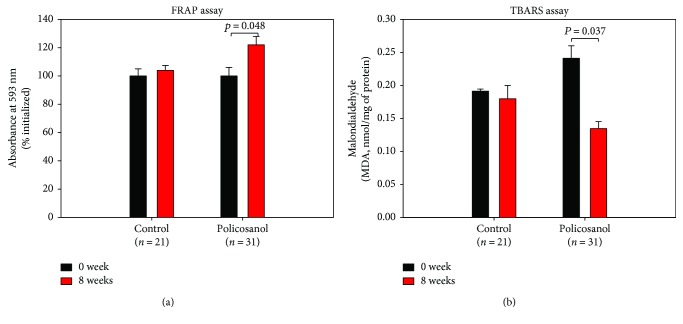
Changes in antioxidant ability and extent of oxidized species in serum upon policosanol consumption (bar represents the standard deviation of the mean). (a) Ferric ion reduction ability of serum (0.05 mL). (b) Determination of oxidized species using the thiobarbituric acid reactive substance method in serum (0.1 mL).

The ferric ion reduction ability of plasma was elevated by 22% in the policosanol group after 8 weeks of consumption, as shown in Figure 2(a), whereas the control group showed no difference over 8 weeks. Malondialdehyde content also significantly decreased up to 50% after 8 weeks of policosanol consumption compared with that at week 0 (Figure 2(b)). The serum uric acid level was reduced by 20% in the policosanol group after 8 weeks of consumption, whereas the control group showed no change (Table 1).

### 3.5. Antioxidative Extent of Lipoproteins

**Figure 3:**
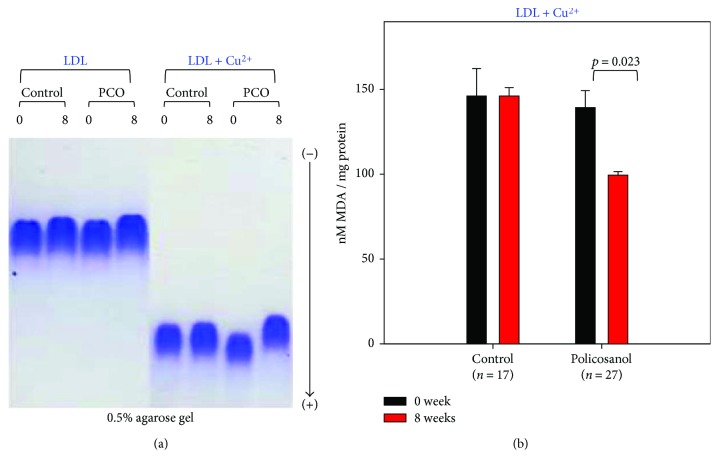
Comparison of LDL oxidation extent during policosanol consumption. (a) Comparison of electromobility of LDL between weeks 0 and 8 with or without cupric ion on a 0.5% agarose gel. (b) Determination of oxidized species using the thiobarbituric acid reactive substance method in LDL (1 mg of protein) in native state at weeks 0 and 8 (bar represents the standard deviation of the mean).

**Figure 4:**
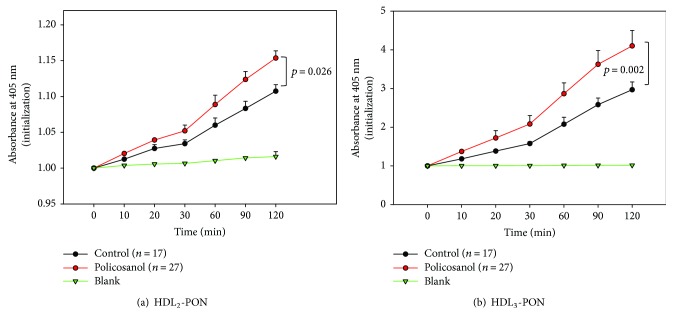
Changes in activity of paraoxonase in HDL at 8 weeks after policosanol consumption. Error bars indicate the SD from three independent experiments with duplicate samples. (a) Equally diluted HDL_2_ (20 mL, 2 mg/mL) was added to 230 mL of paraoxon-ethyl (Sigma Cat. No. D-9286) containing solution (90 mM Tris-HCl/3.6 mM NaCl/2 mM CaCl_2_ [pH 8.5]). (b) Equally diluted HDL_3_ (20 mL, 2 mg/mL) was added to 230 mL of paraoxon-ethyl (Sigma Cat. No. D-9286) containing solution (90 mM Tris-HCl/3.6 mM NaCl/2 mM CaCl_2_ (pH 8.5)) (bar represents the standard deviation of the mean).

After 8 weeks of policosanol consumption, LDL from the policosanol group showed slower electromobility following cupric ion-mediated oxidation and agarose electrophoresis, as shown in Figure 3(a), whereas LDL from the control group showed faster electromobility. Without cupric ion treatment, all LDL showed similar electromobility, although LDL from the policosanol group after 8 weeks showed the slowest electromobility suggesting less production of negatively charged molecules and less fragmentation of apoB in LDL. However, the oxidized LDL moved faster to the cathode position because of the high negative charge and fragmentation of apoB (Figure 3(a)). Quantification of oxidized species using the TBARS method revealed that the policosanol group showed a significantly reduced malondialdehyde (MDA) content (up to 30% less) after 8 weeks, whereas the control group showed no change (Figure 3(b)). After policosanol consumption, PON activities for HDL_2_ and HDL_3_ in the policosanol group were elevated by 14% and 38%, respectively, compared to those of the control group, as shown in Figure 4.

### 3.6. Glycation Extent of Lipoproteins

**Figure 5:**
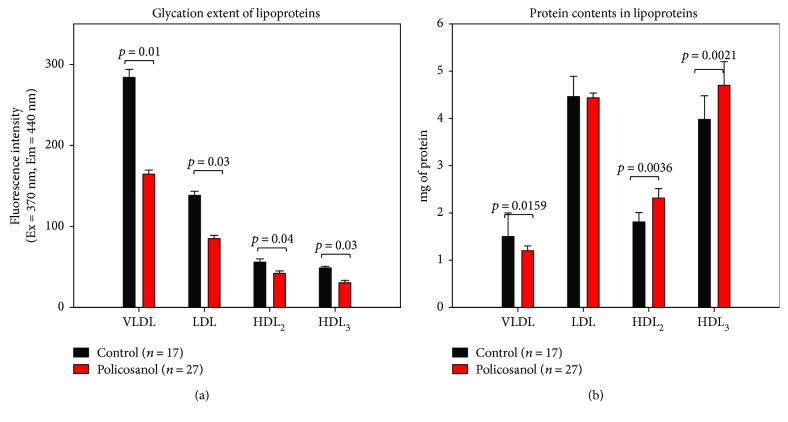
Glycation extent and total amounts of proteins in lipoproteins between groups after 8 weeks of policosanol consumption. (a) Fluorometric determination (Ex = 370 nm, Em = 440 nm) of glycation extent (bar represents the standard deviation). (b) Protein determination of individual lipoproteins (bar represents the standard deviation).

After 8 weeks of policosanol consumption, the policosanol group showed a significantly lowered glycation extent in all lipoprotein fractions, as shown in Figure 5(a). For VLDL and LDL, the policosanol group showed 43% and 39% less production of advanced glycation end products (AGEs) compared to the control group. For HDL_2_ and HDL_3_, the policosanol group showed 25% and 38% less production of AGEs, respectively, than the control group.

Protein content was detected in lipoprotein species, as shown in Figure 5(b). For VLDL, the policosanol group showed 20% less protein content than the control group did, whereas protein content in LDL was similar between the groups. However, protein contents in HDL_2_ and HDL_3_ increased in the policosanol group by 1.3- and 1.2-fold, respectively, compared to the control.

### 3.7. Enhanced Antiatherosclerotic Activity of HDL_3_ in Policosanol Group

**Figure 6:**
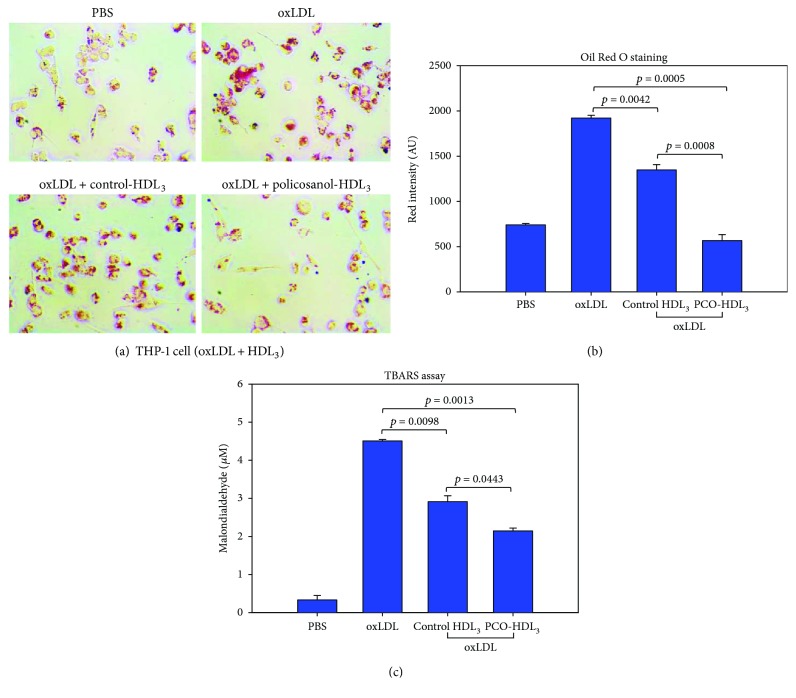
Comparison of oxLDL uptake into macrophages in the presence of HDL_3_ from each group. (a) Inhibition of oxLDL phagocytosis by HDL from each group, as visualized by Oil Red O staining. (b) Quantification of Oil Red O-stained area by computer-assisted morphometry. (c) Quantification of oxidized species in cell culture media using the TBARS method.

As shown in Figures 6(a) and 6(b), oxLDL was easily taken up into macrophages, as evidenced by Oil Red O staining, and HDL_3_ from the control group resulted in 30% inhibition of phagocytosis. Interestingly, HDL_3_ from the policosanol group resulted in 70% reduction of phagocytosis, which was 2.4-fold greater than that of the control group. Quantification of oxidized species in cell culture media showed that oxLDL treatment resulted in the highest level of MDA (around 4.5 *μ*M) in media. HDL_3_ from the policosanol group resulted in the lowest MDA level (around 2.1 *μ*M) in media, whereas control cells showed a 2.9 *μ*M MDA level (Figure 6(c)).

### 3.8. HDL Particle Size and Number

**Figure 7:**
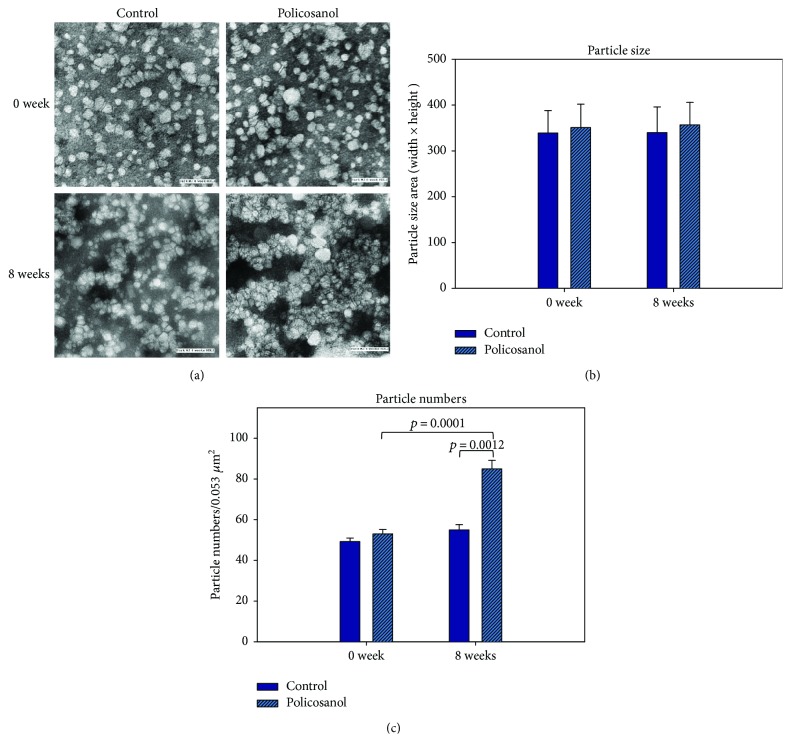
Electron microscopic observation of HDL_2_. (a) Illustrative image of negatively stained high-density lipoprotein 2 (HDL2) from control and policosanol group (electron microscopy). All micrographs are shown at a magnification of 40,000x. Scale bar corresponds to 100 nm. (b) Graphs show measured width and length from 20 particles of HDL. (c) Histogram shows calculated particle numbers per 0.053 *μ*m^2^ area.

TEM image analysis revealed that the control and policosanol groups showed similar particle size after 8 weeks (photos of Figure 7). However, the particle number was significantly elevated up to 1.5-fold in the PCO group compared to the control group (graphs of Figure 7).

### 3.9. Insulin Secretion

**Figure 8:**
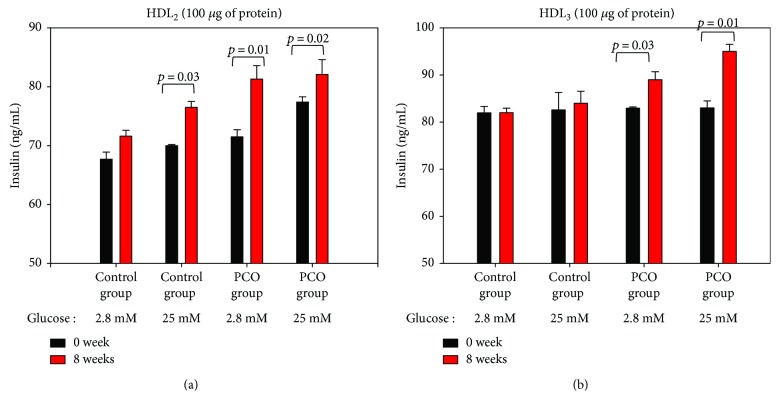
Insulin secretion activity of HDL from each group after 8 weeks of policosanol consumption. Rat insulinoma cells (INS-1) were incubated for 2 hr in the presence of HDL_2_ and HDL_3_ (final 100 *μ*g of protein) at different glucose concentrations (final concentration of 2.8 or 25 mM in culture medium). Insulin levels in medium were quantified using a radioimmunoassay kit. Results are expressed as the mean ± standard deviation (SD) from three independent experiments with duplicate samples.

Under basal and high glucose conditions (final concentrations of 2.8 and 25 mM in media), HDL_2_ from the control group induced 5% and 8% insulin secretion over 8 weeks in rat insulinoma cells (INS-1), as shown in Figure 8. HDL_2_ from the policosanol group caused a significant increase in insulin secretion under basal glucose and high glucose conditions compared to that at week 0.

HDL_3_ from the policosanol group showed a significant enhancement of insulin secretion. In particular, HDL_3_ from the policosanol group caused a 14% increase in secretion compared to that at week 0 under high glucose conditions. Additionally, HDL_3_ from the policosanol group caused a 5% increase in secretion compared to that at week 0 under basal glucose conditions. Moreover, there was no significant difference observed in insulin secretion in control groups under basal and high glucose conditions.

### 3.10. Enhanced Cholesterol Efflux by rHDL-Containing Policosanol

As shown in Supplementary Figure 1A, cholesterol efflux activity increased from 24% to 29% as the policosanol content of rHDL significantly increased from 0.5 to 2.5 *μ*g in the presence of the same amount of rHDL (*p* = 0.03, under 56 *μ*g of apoA-I). Although there was no significant change (*p* = 0.06), 2.5 *μ*g of policosanol in the presence of a lower amount of rHDL (28 *μ*g of apoA-I) more strongly enhanced cholesterol efflux activity than did 0.5 *μ*g of policosanol in the same amount of rHDL (28 *μ*g of apoA-I). However, 0.5 *μ*g of policosanol adequately enhanced cholesterol efflux activity compared with apoA-I alone, and a greater amount of policosanol (2.5 *μ*g) treatment resulted in a higher cholesterol efflux activity with the same amount of apoA-I in rHDL.

In addition to efflux activity, immunodetection revealed that uptake of apoA-I into macrophages was more facilitated (up to 60%) as policosanol content increased with the same amount of apoA-I regardless of cAMP treatment, as shown in Supplementary Figure 1B. The expression level of ABCA1 also increased up to 2-fold upon PCO-rHDL treatment, especially in the presence of cAMP, whereas GAPDH expression as a loading control (total 10 *μ*g of protein from cell lysate) had no effect.

### 3.11. Improved Insulin Secretion by Policosanol

Rat INS-1 cells were incubated with rHDL-containing policosanol under basal (2.8 mM glucose in culture medium) and high glucose conditions (25 mM glucose). After 48 hr of incubation, rHDL-treated cells had insulin secretion levels of 20 ± 2 and 39 ± 4 ng/mL under basal and high glucose conditions, respectively (Supplementary Figure 2). In contrast, cells treated with PCO-rHDL containing 28 *μ*g of apoA-I and policosanol (from 2.5 to 5 *μ*g) showed elevated insulin secretion levels up to 90 and 98 ng/mL, respectively. Cells treated with PCO-rHDL containing 56 *μ*g of apoA-I and policosanol (from 2.5 to 5 *μ*g) showed elevated insulin secretion levels up to 116 and 127 ng/mL under basal and high glucose conditions, respectively. In the presence of the same amount of apoA-I, insulin secretion was elevated up to 1.4-fold depending on the policosanol content in rHDL (Supplementary Figure 2).

## 4. Discussion

In the current study, 8 weeks of policosanol consumption resulted in a reduction in blood pressure and visceral fat amount in healthy female subjects with prehypertension. The lowering effects of policosanol on blood pressure were accompanied by lowering of serum total cholesterol and triglyceride levels as well as increased HDL-C levels via inhibition of serum CETP activity (Table 1). One of the interesting findings of this study is that policosanol could enhance cholesterol efflux in a dose-dependent manner by stimulating the expression of ABCA-1 (Supplementary Figure 1). Cholesterol efflux is a key feature of HDL that exerts regression activity via removal of cholesterol from atherosclerotic plaques in the reverse cholesterol transport pathway. It has been reported that efflux activity is mainly dependent on the configuration of apoA-I [32]. Therefore, the current finding shows that policosanol enhanced cholesterol efflux synergistically with apoA-I. It has been suggested that the apoA-I configuration in discoidal HDL may be important for the recognition of cellular proteins as well as for interactions with specific lipid domains of the cell membrane. Our group previously reported that encapsulation of policosanol in rHDL caused a reduction in *α*-helix content in apoA-I along with an increased exposure of Trp residues [7]. These configurational changes might increase the affinity between apoA-I and the lipid domain of ABCA-1 for enhancement of cholesterol efflux.

Native apoA-I and HDL can stimulate insulin secretion [33] and exert antidiabetic activity, whereas modified apoA-I/HDL cannot. Native reconstituted HDL also displayed insulin secretion activity along with a wound-healing effect. Many studies on patients have reported that policosanol has efficacy in the treatment of hyperlipidemia, diabetes, and hypertension [34, 35], although the detailed molecular mechanism has not been elucidated. As there has been almost no study on the effects of policosanol on healthy subjects with hyperlipidemia and hypertension, this study investigated the efficacy of policosanol in ordinary and healthy subjects with prehypertension.

A recent paper reported that hexacosanol reduces plasma and hepatic cholesterol by activation of adenosine 5′-monophosphate- (AMP-) activated protein kinase (AMPK) and suppression of sterol regulatory element-binding protein-2 in HepG2 and C57BL/6J mice [36]. It has been well known that AMPK activation activities were correlated with increased export of cholesterol and excretion of cholesterol [37]. Recently, AMPK activation enhances antiatherogenic effects of HDL with slightly lowering serum total cholesterol and body weight in apoE^−/−^ mice [38]. Taken together, these papers make agreement that policosanol can enhance HDL functionality via AMPK activation and CETP inhibition.

CETP is an atherogenic factor, which is capable of degenerating HDL functionality and composition. Elevated CETP activity is associated with increased serum TG and TG-enriched LDL levels. We previously reported that policosanol can potently inhibit human CETP *in vitro* [7, 8], similar to MK-0859 (anacetrapib), a CETP inhibitor from Merck (Kenilworth, NJ, USA). Supplementation with policosanol was previously shown to cause significant reduction of CETP activity in zebrafish plasma [8] and human plasma, especially in young and middle-aged healthy male subjects [9]. In the current report, female subjects also showed significant reduction of CETP activity and amount (25%) in serum upon policosanol consumption. It has been well established that impairment of HDL functionality in patients with rheumatoid arthritis is associated with elevation of CETP activity and expression, as in our recent report [39]. Especially in an autoimmune disease state, such as rheumatoid arthritis, arterial stiffness was shown to be positively correlated with elevation of CETP activity [40]. Furthermore, CETP inhibition might be connected to lower visceral fat and antiobesity effect. Since higher CETP activity explains lower HDL-C in obese subjects, it has been suggested that plasma CETP levels may be regulated by the degree of total body fat accumulation [41].

The lipid profile most associated with exacerbation of metabolic syndrome is high TG levels, whereas low HDL-C levels are associated with high risk of insulin resistance [42] and systemic inflammation due to high visceral fat mass [43]. Policosanol consumption reduced the TG/HDL-C ratio and visceral fat amount (Table 1). In the current study, reduction of serum TG level was correlated with reduction of visceral fat mass in the policosanol group, and a Taiwanese study revealed that the serum TG level independently contributes to visceral fat amount [44]. Reduction of serum TG level could cause reduction of visceral fat mass. Nonobese patients with polycystic ovary syndrome show significantly higher serum TG and lower HDL-C levels along with 1.7-fold increased visceral fat thickness [45] despite having normal BMI levels. It has been suggested that visceral fat thickness is negatively correlated with HDL-C level and positively correlated with serum TG level [46]. Further, the TG/HDL ratio can be a predictive marker for the success of antidiabetic medications following weight loss [47].

Systolic and diastolic blood pressures are positively correlated with visceral fat accumulation in premenopausal subjects [48]. These results are in good agreement with our previous report in which male subjects showed reduction of visceral fat mass and blood pressure after 8 weeks of policosanol consumption [9]. Nevertheless, the previous report involved many limitations such as small sample size, weak study design (absence of randomized, double-blinded, and placebo-control), and diagnostic method of blood pressure.

Interestingly, antioxidant ability in plasma was elevated while oxidation of LDL was reduced upon policosanol consumption. PCO-rHDL also showed antioxidant ability against cupric ion treatment, as in our previous report [7, 8]. Resistance of LDL oxidation to cupric ion (Figure 3) is associated with enhancement of HDL-associated paraoxonase activity (Figure 4). Higher paraoxonase activity is also associated with protection of LDL oxidation from lipid peroxidation [49]. The glycation extent of each lipoprotein fraction was reduced by policosanol consumption (Figure 5(a)), although protein content was similar to or higher than that of the control. The antiglycation effect of policosanol consumption (Figure 5) is well correlated with our previous report [7] that PCO-rHDL shows an inhibitory effect *in vitro* against fructose-mediated glycation of HDL. The antiglycation effect is known to be associated with vasorelaxation via improvement of atrial stiffness since AGEs are a proven marker of CVD, diabetes, and hypertension [50]. From a study with Chinese subjects, plasma AGE concentration was found to be positively correlated with pulse wave velocity from the carotid to femoral arteries [51]. More interestingly, the serum TG level was elevated under conditions of high plasma AGE content.

Interestingly, HDL functionality and particle numbers were elevated by policosanol, which is a new finding since there are no agents known to enhance apoA-I expression and HDL quality except curcumin [52]. Although several nutraceuticals have been reported to induce lipid-lowering [53] and arterial hypertension [54], the beneficial functions of lipid-free apoA-I and HDL can be impaired by oxidation and glycation, resulting in amyloid formation and aggregation [55, 56].

PCO-rHDL was taken up more by macrophages via upregulation of ABC-A1 (Supplementary Figure 1). Since dysfunction of ABCA1 is associated with a significant reduction in serum HDL levels, cholesterol efflux ability is positively correlated with apoA-I expression. A few angiotensin receptor blockers such as telmisartan and candesartan are known to interact with ABC transporters [57, 58]. Our current results suggest that policosanol might regulate the expression of apolipoproteins and transporters, which are involved in reverse cholesterol transport. Apart from the functionality of HDL-C and structural changes in lipoproteins, we determine the levels of uric acid and aldosterone levels in both groups. Previous studies have linked the elevation of these biomarkers in blood which may increase the risk of causing hypertension [59–61]. These reports suggested a plausible role of biomarker uric acid and aldosterone levels. Hence, we examine the concentration of these biomarkers before and after policosanol consumption. Our results have demonstrated that the levels of uric acid and aldosterone were significantly reduced after consumption of policosanol for 8 weeks. We did not find any significant difference in the levels of these biomarkers in the placebo group. Clinically, these results imply that enhancement of HDL functionality is well correlated with improvement of blood pressure and visceral fat mass. These results are in good agreement with our previous report in which male subjects showed a reduction of visceral fat mass and blood pressure after 8 weeks of policosanol consumption [9]. The novelty of this study was the study design, notably participant's number, use of placebo group, recruitment of prehypertensive participants (SBP 120–139 mmHg, DBP 80–89 mmHg), three devices used to measuring blood pressure, and homogenous data with a considerable time period of therapy of policosanol. Additionally, this study measured the important biomarkers such as renin and aldosterone, which are previously known parameters that could associate with increased risk of hypertension and CVD. Therefore, the study result could determine the appropriateness and authenticity with respect to the study design, recruitment, and number of participants used in this study.

## 5. Conclusions

The present study tested the effects of policosanol on biomarkers of HDL functionality, including cellular cholesterol efflux, insulin secretion, CETP activity, paraoxonase activity, and apoA-I level, after 8 weeks of policosanol consumption. Improvement of HDL functionality was associated with lowered blood pressure and inhibition of CETP activity in female prehypertension subjects.

## Figures and Tables

**Table 1:** Change of blood pressure and plasma profile after 8 weeks consumption.

	Placebo (*n* = 21)		Policosanol (*n* = 31)	
Age	31 ± 16		31 ± 15	
	Week 0	Week 8	Week 0	Week 8
*Body composition*				
BMI	21 ± 3	21 ± 3	21 ± 4	21 ± 4
Total body fat (kg)	13.5 ± 3	13.1 ± 3	15.2 ± 4	13.8 ± 4^∗^
Percentage of body fat (%)	24 ± 5	23 ± 6	25 ± 6	23 ± 5
Subcutaneous fat (kg)	11.9 ± 2.1	11.7 ± 2.4	13.6 ± 3.3	12.5 ± 3.0
Visceral fat (kg)	1.3 ± 0.4	1.4 ± 0.4	1.5 ± 0.5	1.2 ± 0.4^∗^
*Blood pressure (mmHg)*				
SphygmoCor XCEL				
Systolic	133 ± 14	126 ± 8	131 ± 10	118 ± 14
Diastolic	87 ± 10	85 ± 8	82 ± 9	75 ± 7
Omron blood pressure monitor				
Systolic	131 ± 16	129 ± 9	130 ± 10	119 ± 8
Diastolic	86 ± 11	85 ± 7	83 ± 8	67 ± 7^∗^
Mercury sphygmomanometer				
Systolic	131 ± 9	125 ± 8	129 ± 8	116 ± 12^∗^
Diastolic	84 ± 6	83 ± 5	84 ± 8	72 ± 8^∗^
Average blood pressure				
Systolic	132 ± 12	127 ± 7	130 ± 7	117 ± 14^∗^
Diastolic	86 ± 9	84 ± 6	83 ± 8	72 ± 8^∗^
Augmentation index (AI)	17 ± 3	16 ± 3	16 ± 3	7 ± 1^∗^
Augmentation pressure (AP)	7 ± 1	5 ± 1	7 ± 2	2 ± 0^∗^
*Plasma profile*				
TC (mg/dL)	195 ± 22	201 ± 21	180 ± 14	146 ± 10^∗^
TG (mg/dL)	84 ± 17	92 ± 20	83 ± 16	72 ± 12^∗^
HDL-C (mg/dL)	42 ± 3	45 ± 4	42 ± 4	53 ± 8^∗∗^
%HDL-C	21 ± 3	22 ± 3	23 ± 2	36 ± 4^∗∗^
TG/HDL-C	2.0 ± 0.6	2.1 ± 0.6	2.0 ± 0.2	1.4 ± 0.1^∗^
Calculated LDL-C (mg/dL)	138 ± 22	137 ± 20	125 ± 11	81 ± 7^∗^
Glucose (mg/dL)	87 ± 5	89 ± 5	91 ± 6	83 ± 5
CETP activity (% CE transfer)	38 ± 4	40 ± 4	39 ± 5	31 ± 4^∗^
CETP amount (*μ*g/mL)	1.9 ± 0.2	1.9 ± 0.2	2.0 ± 0.2	1.5 ± 0.1
Uric acid (mg/dL)	6.7 ± 1.4	6.5 ± 2.4	6.6 ± 0.8	5.3 ± 1.2^∗^
Aldosterone (ng/dL)	19 ± 7	25 ± 8	38 ± 10	23 ± 6^∗^

AU: arbitrary unit; BP: blood pressure; BMI: body mass index; CETP: cholesteryl ester transfer protein; HDL-C: high-density lipoprotein cholesterol; TC: total cholesterol; TG: triglyceride; ^∗^ *p* < 0.05; versus 0–8 weeks in each group.

## Abbreviations

BP: blood pressure
BMI: body mass index
CETP: cholesteryl ester transfer protein
TC: total cholesterol
TG: triglyceride
TP: total protein
HDL-C: high-density lipoprotein-cholesterol
HDL: high-density lipoprotein
LDL: low-density lipoprotein
VLDL: very low-density lipoprotein.

## Supplementary Materials

Supplementary Materials Supplementary Figure 1: (A) cholesterol efflux activity of rHDL-containing apoA-I and policosanol. Macrophages were treated with radiolabeled cholesterol. The isotope amount of cholesterol taken up into cells was quantified, as detailed in the text; (B) immunodetection of apoA-I and ABCA-1 in cell lysate after treatment with rHDL. BI: band intensity. Supplementary Figure 2: insulin secretion activity of rHDL-containing policosanol. Rat insulinoma cells (INS-1) were incubated for 2 hr in the presence of rHDL with or without policosanol (final concentration of 2.5–5 mg in media) at different glucose concentrations (final concentration of 2.8 or 25 mM in culture medium). Insulin levels in medium were quantified using a radioimmunoassay kit. Results are expressed as the mean ± standard deviation (SD) from three independent experiments with duplicate samples.
