# Supplementary material for: Consumption of Cuban Policosanol Improves Blood Pressure and Lipid Profile via Enhancement of HDL Functionality in Healthy Women Subjects: Randomized, Double-Blinded, and Placebo-Controlled Study
Source: Oxid Med Cell Longev. 2018 Apr 16;2018:4809525. doi: 10.1155/2018/4809525 (PMC5944267; doi:10.1155/2018/4809525)
Supplement: Supplementary Materials — Supplementary Figure 1: (A) cholesterol efflux activity of rHDL-containing apoA-I and policosanol. Macrophages were treated with radiolabeled cholesterol. The isotope amount of cholesterol taken up into cells was quantified, as detailed in the text; (B) immunodetection of apoA-I and ABCA-1 in cell lysate after treatment with rHDL. BI: band intensity. Supplementary Figure 2: insulin secretion activity of rHDL-containing policosanol. Rat insulinoma cells (INS-1) were incubated for 2 hr in the presence of rHDL with or without policosanol (final concentration of 2.5–5 mg in media) at different glucose concentrations (final concentration of 2.8 or 25 mM in culture medium). Insulin levels in medium were quantified using a radioimmunoassay kit. Results are expressed as the mean ± standard deviation (SD) from three independent experiments with duplicate samples. [file 4809525.f1.pdf]

Suppl. Fig. 1A

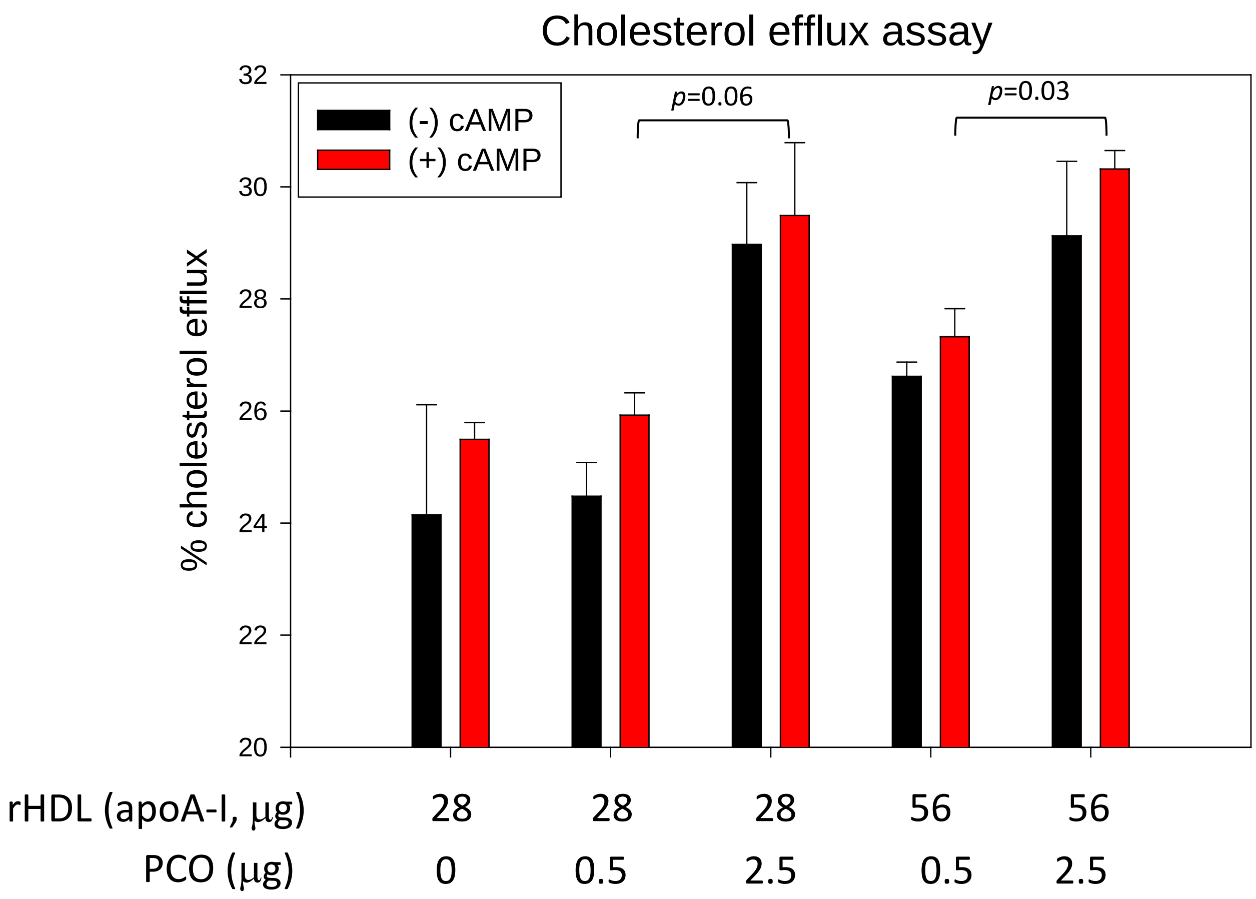

Cholesterol efflux activity of rHDL containing apoA-I and policosanol.
Macrophages were treated with radiolabeled cholesterol.
The isotope amount of cholesterol taken up into cells was quantified, as detailed in the text.

Suppl. Fig. 1B

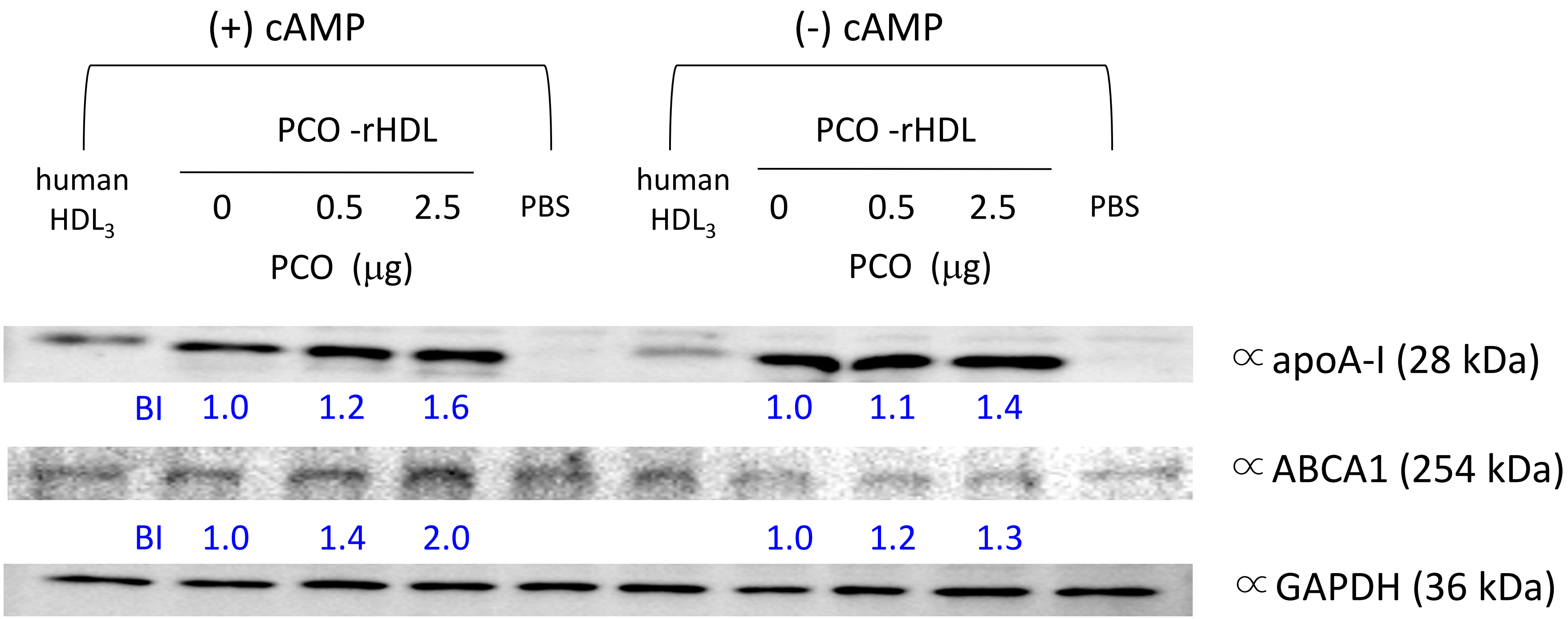

Immunodetection of apoA-I and ABCA-1 in cell lysate after treatment with rHDL. BI, band intensity.

Suppl. Fig. 2

Insulin secretion (INS-1 cell)

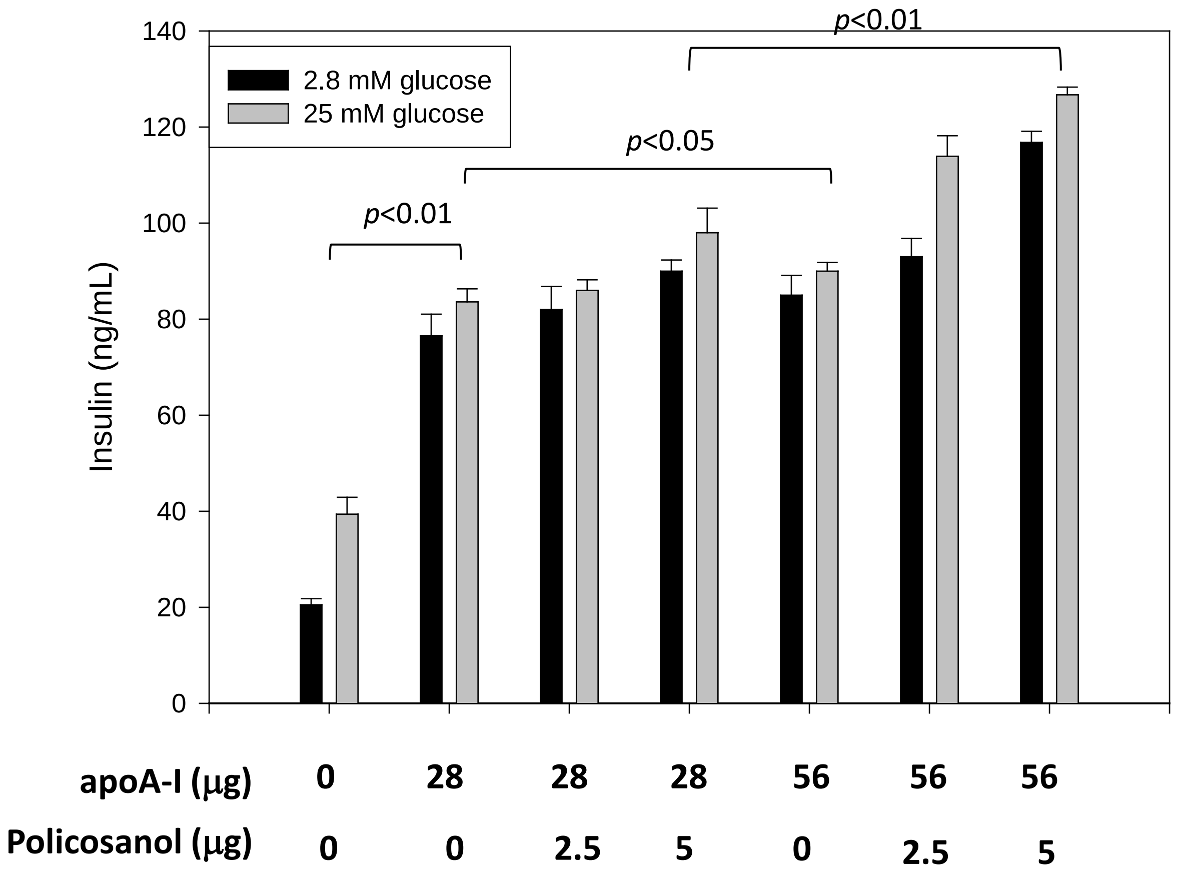

Insulin secretion activity of rHDL containing policosanol.
Rat insulinoma cells (INS-1) were incubated for 2 hrs in the presence of rHDL with or without policosanol (final concentration of 2.5-5 mg in media) at different glucose concentrations (final concentration of 2.8 or 25 mM in culture medium). Insulin levels in medium were quantified using a radioimmunoassay kit. Results are expressed as the mean – standard deviation (SD) from three independent experiments with duplicate samples.
